# Postoperative management following osteotomies around the knee: a narrative review

**DOI:** 10.1530/EOR-23-0153

**Published:** 2024-07-01

**Authors:** Felix Christoph Finger, Steffen Schröter, Christoph Ihle, Moritz Herbst, Tina Histing, Marc-Daniel Ahrend

**Affiliations:** 1 BG Klinik Tübingen, Department of Traumatology and Reconstructive Surgery, Eberhard Karls University of Tübingen, Tübingen, Germany; 2 Diakonie Klinikum Jung-Stilling GmbH, Department of Trauma and Reconstructive Surgery, Siegen, Germany; 3 Osteotomie Komitee der Deutschen Knie Gesellschaft (DKG), Munich, Germany; 4 AO Research Institute Davos, Davos, Switzerland

**Keywords:** postoperative management, knee osteotomy, rehabilitation, HTO

## Abstract

- The present narrative review provides a summary of postoperative therapy modalities and their effectiveness following osteotomies around the knee.
- The topics that are discussed in the scientific discourse include support of cartilage cell regeneration, pain management, drainage insertion, tourniquet use, pharmacological and mechanical thromboembolism prophylaxis, weight-bearing protocols and bone consolidation.
- There is evidence for the use of pharmacological thromboembolism prophylaxis and weight-bearing protocols.
- A standardized postoperative treatment concept following osteotomies around the knee cannot be derived due to lack of evidence for the other topics in current literature.

## Introduction

Patients expectations on the postoperative outcome after knee osteotomy are high, especially with regard to pain reduction, regaining knee functionality and return to work (1). The postoperative period contributes essentially to the overall treatment success, as pain reduction, early mobilization, reduction of swelling, prevention of complications and consequently faster return to work and sports may be influenced by the postoperative treatment. However, the postoperative period following knee osteotomies is markedly less represented in the scientific discourse and less discussed than preoperative planning and surgical techniques. This is reflected by many publications about the pre- and intraoperative period, compared to studies about the postoperative period. The issues return to work and return to sports are exceptions and are well represented in systematic reviews and meta-analyses (2, 3, 4, 5, 6).

The aim of this narrative review is therefore to summarize and present current literature regarding therapeutic options and their effectiveness in the postoperative treatment following osteotomies around the knee.

The presented results should be verified by each reader himself regarding suitability and applicability for clinical practice according to availability and regional healthcare system characteristics.

## Methods

**Figure 1:**
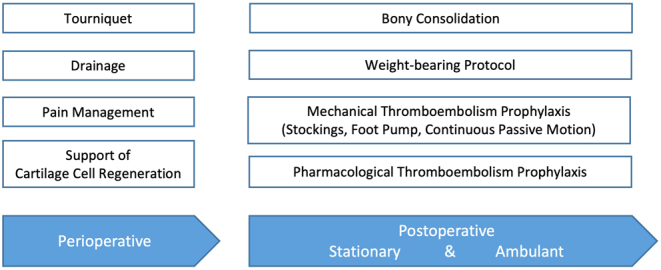
Selected topics of the postoperative treatment concept following osteotomies around the knee.

The current guideline on postoperative treatment concepts of the German Society for Orthopaedics and Trauma Surgery frames general goals of postoperative treatment, as well as surgery-specific treatment recommendations after open-wedge high tibial osteotomy (HTO) (7). Referring to the recommendations of the postoperative treatment guideline, Fig. 1 shows an overview of topics in the postoperative treatment following osteotomies around the knee.

For each recommendation, a literature research was conducted in the Medline database on its effectiveness and proven benefit. In addition, a literature search with the terms ‘osteotomy’, ‘rehabilitation’ and ‘knee’ was carried out. References of relevant articles were screened for further suitable studies. Included studies were assigned to the thematic groups shown in Fig. 1 and summarized subsequently.

## Results

### Perioperative management

#### Support of cartilage cell regeneration

It is the aim of knee osteotomies to change the pressure distribution in the knee joint to enable regeneration or preservation of cartilage tissue. The injection of mesenchymal stem cells (MSCs) to support cartilage regeneration was analyzed by Jin *et al.* in a systematic review (8). It showed that MSC injection following HTO results in significant higher Lysholm scores (mean difference (MD) = 2.55; 95% CI: 0.7–4.4) and HSS scores (MD = 4.33; 95% CI: 0.99–7.68) compared to a control group. No significant differences were found for the IKDC score (MD = 4.81; *P* = 0.06) and the KOOS subscales pain (MD = 4.15; *P* = 0.15) and symptoms (MD = 3.53; *P* = 0.32). Additionally, two of six included studies (*n* = 174) showed a superior cartilage regeneration in a second arthroscopy as measured by the International Cartilage Regeneration & Joint Preservation Society (ICRS) scale (significantly more proportions grade 1 (odds ratio (OR) = 4.13; 95% CI: 1.12–15.26) and grade 2 (OR = 2.38; 95% CI: 1.18–4.80) at the medial femoral condyle) (8). However, the duration between index surgery and re-arthroscopy was not reported. The comparability of the included studies of the review is difficult due to the variation of control groups. The following comparisons were made: HTO with adipose stem cells (aMSCs) vs HTO only (9), HTO with aMSC and platelet-rich-plasma (PRP) vs HTO and PRP (10), HTO and human umbilical cord blood vs HTO and bone marrow aspirate concentrate (11, 12), HTO and human umbilical cord blood vs HTO and micro-fracture (13), HTO and MSCs from bone marrow vs HTO only (14).

Based on the results of Jin *et al.* (8) the benefit of intra-articular MSC injections concerning cartilage regeneration and functional outcomes after knee osteotomies should be further examined. MSC injections may have positive benefits for the treatment outcome and therefore further randomized controlled trials (RCTs) are necessary. Two additional studies on MSC injections were published after publication of the systematic review of Jin *et al.* They show favorable trends for cartilage cell regeneration, but its generalizability is limited due to small sample sizes (*n* = 20 (15) and *n* = 12 (16)).

#### Pain management

Postoperative pain reduction can be achieved by regional anesthesia. A randomized, placebo-controlled study by Sim *et al.* investigated the effect of adductor-canal nerve block (20 mL levobupivacaine single-shot) on pain perception and range of motion (ROM), postoperative opioid consumption, m. quadriceps femoris strength and occurrence of complications following medial open-wedge HTO (17). To prevent PONV (postoperative nausea and vomiting), all patients received 4 mg dexamethasone. Pehora *et al.* showed in their Cochrane Review, that Dexamethasone administration can prolong the duration of effectiveness of nerve blocks at the upper extremity. There is not enough evidence for the prolonging effect for nerve blocks at the lower extremity (18). Sim *et al.* compared an intervention group (adductor canal block = ACB; *n* = 19) with a placebo group (saline solution; *n* = 16) (17). The postoperative opioid consumption and pain perception was significantly lower for the first 12 h after surgery in the intervention group compared to the placebo group (16.7% vs 70% for opioid consumption, values not reported for pain perception). Likely, the percentage of patients who received more than 5 opioid infusions during the first 72 h after surgery was significantly lower in the ACB group (8%) compared to the placebo group (50%). Strength of m. quadriceps femoris did not differ significantly after 72 h, 2 weeks and 3 months. No significant difference was found for the point of time at which straight leg rising could be performed (23.5 ± 17.7 h ACB vs 27.6 ± 11.4 h placebo group), for ROM and pain perception from 12 h post surgery onwards. There were no local complications in both groups. Sim *et al.* state that retained strength of m. quadriceps femoris combined with proven analgesic effect of the ACB is beneficial for early postoperative mobilization (17). A direct comparison of n. femoralis and adductor-canal blocks was not performed in the study. Ren *et al.* showed in a randomized controlled trial, that n. femoralis blocks (20 mL 0.25% ropivacaine) reduce pain perception 12 h after medial open-wedge HTO significantly (visual analogue scale = VAS 3.5 ± 1.0 vs 4.7 ± 1.1 for control group without block) (19). Epidural anesthesia was administered both in the nerve block group (*n* = 20) and the control group (*n* = 21). No difference for m. quadriceps femoris strength was noted after 24 h and 48 h post surgery. There were no regional anesthesia associated falls during postoperative treatment, but weight-bearing protocols were not published (19). Further studies, such as on the effect of nerve blocks following HTO under general anesthesia are not available. It is important to consider the risk of falling under peripheral nerve blocks against the benefit for pain relief, especially when following early postoperative mobilization protocols. Patients should be extensively instructed and initially supported by physiotherapists. Knee orthoses in full extension should be used as long as nerve blocks are active. According to the opinion of the authors, the potentially increased risk of falling must be carefully considered against the advantages. Especially if patient compliance is presumably low and rapid self-initiated mobilization (e.g. smoking) is likable, regional anesthetic procedures should be avoided.

#### Application of drainages

Application of drainages is controversially discussed. Li *et al.* conducted a prospective, randomized controlled trial on the effect of drainage application on postoperative pain, blood loss and occurrence of complications following HTO (20). The osteotomy gap was filled with beta-tricalcium phosphate bone graft material. It should be noted that the use of substitution material can lead to increased pseudarthrosis rates (21, 22) and shows no superiority for clinical scores (23) and is therefore not recommended in the opinion of the working group of this review.

In the study by Li *et al.*, the drainage was opened 2 h after surgery and removed on the first postoperative day (20). Whether a negative pressure was set on the drainage was not described. Li *et al.* showed that the group with drainage (*n* = 40) achieved significantly lower VAS scores, reduced lower limb swelling and a better ROM in the short-term postoperative period (days 1–5) compared to a control group without drainage (*n* = 40). In addition, there were significantly fewer wound complications and less wound secretion. Total blood loss during the postoperative period and hemoglobin levels showed no significant difference. Three months after surgery, no differences were found for VAS and the Hospital for Special Surgery (HSS) Knee Rating Scale (20). Li *et al.* therefore suggest drainage application following HTO. The influence of drainage application on the ossification of the osteotomy gap was not discussed. In particular, when the osteotomy gap is not filled, the insertion of a drainage must be critically questioned, as ossification could be impaired by delayed formation of the blood coagulum in the osteotomy gap. This should be investigated in further publications, as evidence is missing in current literature. Likewise, differences in benefits of drainage application for open versus closed wedge osteotomies should be evaluated.

#### Tourniquet use

Though the application of tourniquets for osteotomies is being discussed more frequently, there is little scientific background. Li *et al.* performed a prospective, randomized controlled trial on the use of tourniquets for medial open wedge high tibial osteotomies (24). Groups were distinguished by tourniquet use only for the arthroscopic exploration (*n* = 45) or for the complete HTO procedure (*n* = 45). They compared peri-operative complications (deep vein thrombosis, pulmonary embolisms, neurovascular events, lateral hinge fractures, wound complications, blood transfusions, readmissions) as well as intra- and post-operative parameters, in detail arthroscopy time, surgery time, length of incision, hospital stay duration, postoperative hemoglobin and hematocrit levels, volume of drainage, total blood loss and required morphine medication. Moreover, postoperative ROM, VAS scores and calf circumference was assessed 1, 3 and 5 days and 3 months post operation. In the group not using a tourniquet for HTO, statistical significance was present for less tight-related complications, shorter hospital stays and fewer usage of morphine medication, as well as for superior ROM in the first 5 postoperative days. Moreover, tourniquet use did not reduce surgery time, intra-operative complications or postoperative blood loss. Li *et al.* therefore do not recommend tourniquet use for HTO procedures (24). Similar results were published by Wang *et al.* in their retrospective study on tourniquet use for HTO (25). They showed that not using a tourniquet led to higher intraoperative blood loss, but total blood loss was not significantly different. Tourniquet use led to significantly inferior scores regarding pain levels and early postoperative ROM in the first two days. Length of hospital stay, complication rates and short-term outcomes 3 months after surgery showed no significant differences (25). Motycka *et al.* examined the incidence of DVT with (*n* = 37) and without the use of a tourniquet (*n* = 28) during HTO and could not find a statistical difference with an average incidence of 10.8% (26). Beyond these studies there is little scientific background on the pros and cons of using tourniquets during osteotomies around the knee. The ongoing discussion whether to use a tourniquet or not is quite likely influenced by the growing evidence regarding tourniquet use in total knee arthroplasty (TKA). Several meta-analyses and systematic reviews for TKA procedures with varying conclusions for tourniquet use are available. Conclusions range from equal clinical results and length of hospital stay (27) to inferior results for postoperative pain, knee function, ROM, swelling and length of hospital stay while having no influence on total blood loss (28) to suggestions that tourniquet use is probably associated with an increased risk for serious adverse events (29). Regarding osteotomies, serious complications such as vascular injuries might be detected and treated immediately when not using a tourniquet. Concerning intra- and post-operative bleeding management there is growing evidence for the use of tranexamic acid (TXA) for osteotomies. A systematic review by Bierke *et al.* confirmed a lower hemoglobin decrease, less total blood loss and lower drainage volume when using TXA during osteotomies at knee level, compared to control groups not using TXA (30). Similar results are published in several other systematic reviews and meta-analyses focusing on HTO (31, 32, 33, 34). A current trend to perform osteotomies without tourniquet use may be deduced from literature on TKA procedures but further high-quality studies must be carried out for osteotomy procedures to provide evidence for this topic.

### Postoperative management

#### Pharmacological thromboembolism prophylaxis

The S3-guideline ‘prophylaxis of venous thromboembolism’ (German association of scientific medical professional societies) in the latest, but currently under revision version from 2015, describes a strong recommendation for pharmacological prophylaxis with low molecular weight heparins or fondaparinux following osteotomies around the knee (35). However, the need for postoperative anticoagulation to prevent thrombosis is controversially discussed for certain patient groups. The incidence of deep vein thrombosis (DVT) varies between studies and depends on the diagnostics used. Onishi *et al.* compared the incidences of DVT in medial open wedge HTO (MOWHTO), lateral closed wedge HTO (LCWHTO) and double level osteotomies (DLO) (36). The patients were examined postoperatively for DVT with ultrasound. Incidences of 11.9% for MOWHTO, 22.6% for LCWHTO and 6.8% for DLO were observed. The high incidence rates, although all patients received chemoprophylaxis with edoxaban, can most likely be explained by screening all patients with ultrasound to detect a DVT. Onishi *et al.* could not determine how many of the detected DVTs were symptomatic. Symptomatic pulmonary embolism (PE) did not occur. The incidence of DVT was significantly higher in the LCWHTO (22.6%) than in the MOWHTO (11.9%) and DLO (6.8%) groups (36). Similar results are shown by the study of Kim *et al.* (37). In postoperative CT scans, DVT was detected in 14.9% in a placebo group and 10.6% in an intervention group receiving 2.5 mg fondaparinux. No patient developed any symptoms or PE. Kubota *et al.* were able to detect DVT postoperatively with sonography in 25.5% of a cohort of 137 patients under thromboembolism prophylaxis with edoxaban (38). There was also no symptomatic DVT and no PE.

In a systematic review, Noyes *et al.* investigated the incidence of deep vein thrombosis and pulmonary embolism after HTO, as well as the need for postoperative thromboembolism prophylaxis (39). The incidence rates for DVT and PE were 1.24% and 0.11% for a total of 7085 knees. Compared to the results presented above, the incidence rates are considerably lower, since postoperative radiological examinations for DVT diagnostics were not defined as an inclusion criterion in the review. The relative risk of DVT was higher in patients with pharmacological prophylaxis (anticoagulants or platelet inhibition) than in patients who did not receive pharmacological prophylaxis (2.0% vs 1.1%, *P* = 0.02). Noyes *et al.* concluded that the use of pharmacological thromboembolism prophylaxis in patients at normal risk is questionable (39).

In common practice, it is difficult to dispense with the pharmacological thromboembolism prophylaxis. However, based on the results of Noyes *et al.*, the indication for pharmacological thromboembolism prophylaxis for patients with a low-risk profile should be critically reconsidered. In principle, the patient- and surgery-specific bleeding risk should be taken into account when using anticoagulants, as well as the HIT II (heparin-induced thrombocytopenia type II) risk, especially when using unfractionated heparin and the risk of osteoporosis in long-term use (35). Therefore, a classification of patients into risk groups must be comprehensible and feasible. Noyes *et al.* describe former DVT in personal or family history, obesity, use of oral contraceptives and hereditary diseases such as factor V disease mutation, prothrombin G20210 mutation, protein C, protein S and antithrombin-III deficiencies as significant risk factors for DVT (39). In summary, thrombosis after knee osteotomies is often asymptomatic and therefore remains undetected or is only detected by imaging diagnostics. Whether the long-term outcome for patients is impaired by asymptomatic DVT is not clear. Kubota *et al.* found significantly lower values for the single-leg standing test postoperatively in the group of patients with verified asymptomatic DVT, compared to the group without DVT (7.0 ± 9.4 s vs 15.4 ± 12.9 s) (38).

#### Mechanical thromboembolism prophylaxis

Thromboembolism prophylaxis is supplemented by numerous mechanical treatments. The current postoperative treatment guideline of the German Society for Orthopaedics and Traumatology recommend the use of continuous passive motion devices (CPM), wearing of medical compression stockings (MCS) and the use of intermittent pneumatic compression devices (IPC) from the first postoperative day after MOWHTO (7). Goosmann *et al.* conducted a literature review on the effectiveness of CPM after knee surgery (40). No controlled studies were found for CPM after knee osteotomies. For CPM therapy after anterior cruciate ligament (ACL) replacement, two reviews discussed in the work of Goosmann *et al.* showed no conclusive superiority of CPM application over physiotherapy (40). A meta-analysis on the effectiveness of CPM therapy after ACL reconstruction conducted by Jaspers *et al.* showed improved knee flexion in the 3rd–7th postoperative days, as well as in the 3rd–6th postoperative weeks, compared to control groups without CPM (41). Knee extension was not significantly improved. Furthermore, patients with CPM therapy required significantly less pain medication on the 1st and 2nd postoperative day (41). Two recently published meta-analyses by Yang *et al.* (42) and Yang *et al.* (43) on the effectiveness of CPM after implantation of knee endoprostheses do not describe any treatment effects with regard to postoperative mobility, knee function and pain symptoms. The use of CPM after knee osteotomies cannot be evaluated on a scientific basis due to the limited literature currently available. As the effectiveness of CPM therapy is only partially proven for other knee surgeries, its benefit should be examined especially for knee osteotomies and weighed against the resulting additional costs.

In a consensus paper of the ‘Asia-Pacific Region Venous Thromboembolism Consensus Group’ from 2021, intermittent pneumatic compression devices, venous foot pumps and medical compression stockings are described as evidence-based effective methods for thromboembolism prophylaxis (44). In a systematic review, Pavon *et al.* investigated the effectiveness of different IPC systems in patients after surgeries with a high risk for thromboembolism (45). The results show that the combination of pharmacological prophylaxis and IPC systems can reduce the occurrence of thromboembolism events. For the use of IPC versus pharmacological prophylaxis after knee or hip arthroplasty, Pavon *et al.* could not find a significant difference in the frequency of thromboembolism events. A meta-analysis evaluating postoperative bleeding risk under IPC versus anticoagulation showed a non-significant tendency towards slightly lower bleeding risk under IPC (relative risk of large bleeding 0.33; 95% CI: 0.07–1.51) for the IPC group vs group with anticoagulation). The ten included RCTs that compared IPC with anticoagulation were all performed after hip (HA) or knee arthroplasty (KA). None of the studies investigated the effect following knee osteotomies (45). Kim *et al.* conducted a study on the effectiveness of IPC vs chemoprophylaxis after KA in 1259 knees. Significantly more DVTs occurred with IPC than with chemoprophylaxis (11.3% vs 6.3% of cohorts) (46). A comparison between the IPC cohort and a control group that received neither chemoprophylaxis nor IPC found no significant difference (11.3% vs 14.8% of the cohorts). There was no major postoperative bleeding in all groups (46). The results for the effectiveness of IPC after major surgeries on hip and knee are inconclusive. The effectiveness of IPC after knee osteotomies should therefore be investigated in high-quality studies.

Sachdeva *et al.* conducted a Cochrane Review on the effectiveness of medical compression stockings (MCSs) for DVT prophylaxis (47). In a meta-analysis of 19 RCTs with surgical interventions (10 abdominal, 6 orthopedic, 1 each neurosurgical, gynecological and cardiological), a significantly lower incidence of DVTs was found in the MCS group (134 of 1365; 9.8%) compared to the control group (282 of 1328; 21.2%) with an OR of 0.35 (95% CI: 0.28–0.44). MCS were worn from the time of hospitalization until full mobilization or discharge. The MCS group received inconsistently additional measures for thromboembolism prophylaxis. The control group received either no thromboembolism prophylaxis or chemotherapy only (47). Shalhoub *et al.* published an RCT on incidence rates in patient cohorts with moderate or high risk of thromboembolism who received postoperatively low molecular weight heparins only or in combination with MCS (48). Follow-up examination regarding incidence of thromboembolism was performed up to 90 days after surgery. In the group without MCS, thromboembolism occurred in 16 out of 937 cases (1.7%), compared to 13 out of 921 (1.4%) cases in the group with MCS. A risk difference of 0.30% (95% CI: −0.65–1.26%) and the analysis of the confidence interval showed no inferiority of the sole administration of low weight molecular heparins (*P* < 0.001). Shalhoub *et al.* conclude that MCS may be unnecessary for most elective surgeries. The relevance of these results for use after knee osteotomies is limited, as the proportion of orthopedic operations was less than 2% in both groups. About half of the operations performed were gastrointestinal and gynecological interventions (48). According to the S3-guideline prophylaxis of venous thromboembolism, wearing of MCS is not necessary when pharmacological thromboembolism prophylaxis is used (35). In a randomized, controlled pilot study on the use of MCS after minor arthroscopic procedures (meniscectomy, cartilage surgery, partial synovectomy), Tischer *et al.* found significantly less swelling at the calf and knee on the 10th postoperative day. The MCS were worn for 10 days in the intervention group and 1 day in the control group (49). For the use of MCS after knee arthroplasty, Munk *et al.* showed no efficacy with regard to postoperative swelling, knee flexion, Oxford knee score and pain (50). With regard to thromboembolism prophylaxis, wearing of MCS after knee osteotomies can be dispensed with in the case pharmacological prophylaxis is used. The findings on swelling reduction should be evaluated more closely. Modern measurement methods for determining swelling should be used and the results considered in a clinical context. Due to the potential for the reduction of swelling after other knee surgeries, this effect should also be investigated for wearing MCS following knee osteotomies.

#### Support of bone consolidation

In a randomized, placebo-controlled, double-blind study, Ziegler *et al.* investigated the influence of ultra-low-frequency pulsed electromagnetic fields (16 Hz, 6-282 μT) on bone consolidation after HTO in patients over 40 years of age (51). Seventy-four patients were evaluated with regard to bone consolidation and blood serum markers. It was shown that bone consolidation was significantly accelerated in a subcohort of patients over 50 years of age compared to the placebo group. In line with this, earlier increases in BAP (bone-specific alkaline phosphatase) serum levels, a marker of osteoblast activity, were detected in the intervention group (51). In contrast, Goshima *et al.* showed in a recently published study on 136 knees that low-intensity pulsed ultrasound (LIPUS) does not promote bone healing after opening HTO (52). The study found no significant differences in bone consolidation of the osteotomy gap at 6 weeks, as well as 3, 6 and 12 months between the intervention group and a control group. In general, the effectiveness of LIPUS application is unclear. In a meta-analysis published in 2017 by Schandelmaier *et al.*, RCTs were analyzed that investigated the use of LIPUS in various types of fractures or osteotomies (53). The evaluation of the high-quality RCTs showed no positive effect of LIPUS on the outcome parameters. These studies focused on patient cohorts with acute fractures. Studies related to knee osteotomies were not included in the meta-analysis (53).

In summary, the effectiveness of a postoperative therapy to promote bone consolidation after knee osteotomies is not sufficiently clarified and therefore needs further research. However, for patients with limited bone metabolism such as elderly patients, smokers, patients with diabetes or poor bone quality, according to the results of Ziegler *et al.*, a supportive therapy for ossification can be justified (51). Especially since increased complication rates have been described for these patient groups (54, 55, 56). This is also conceivable in the case of postoperatively proven hinge fractures with higher rates of pseudarthrosis (57, 58). Furthermore, it should be analyzed whether ceasing nicotine consumption has a direct effect on bone consolidation.

#### Weight-bearing protocol

It is important to consider the type of osteotomy, the choice of implants, as well as the management of the osteotomy gap filling when interpreting study results. Angular-stable plate fixator systems such as the TomoFix plate for bridging the osteotomy gap are considered particularly stable (59) and have already been tested for early functional loading (60).

Schröter *et al.* conducted a prospective, randomized controlled study on 120 patients treated with MOWHTO, TomoFix plate fixation and without filling the osteotomy gap. In the intervention group full weight-bearing was allowed 11 days after MOWHTO, in the control group after 6 weeks (61). Until full weight-bearing was allowed, partial weight-bearing with 20 kg using crutches was carried out. It was shown that the Lysholm and Lequesne scores increased significantly more than those in the control group 6 months postoperatively. After 18 months, equally good clinical scores were achieved in both groups. Schröter *et al.* conclude that functional full weight-bearing in the early postoperative period can be reasonable and beneficial (61).

Similar findings can be drawn from the meta-analysis of van Haeringen *et al.* (62). The aforementioned study by Schröter *et al.* (61) was included in the meta-analysis. Van Haeringen *et al.* analyzed RCTs for the comparison between early (0–11 days post operation) and delayed full weight-bearing (6–8 weeks post operation) after HTO. The IKDC score at 1 year, as well as activity at 3 months, showed no significant difference between early and delayed weight-bearing protocols. There were no increased complications after early weight-bearing when using angular-stable plate fixation. It is concluded that under the conditions of angular-stable plate fixation, early postoperative full weight-bearing following MOWHTO is safely feasible (62). A meta-analysis by Lee *et al.* confirmed that early full weight-bearing about 2 weeks after open-wedge HTO is feasible without an increased risk of complications when using an angular-stable plate fixation (63).

In summary, early functional weight-bearing after open-wedge HTO seems to be possible without an increased risk of complications, taking into account the principles of angular-stable plate fixation. With regard to postoperative weight-bearing protocols when using other fixation principles or osteotomies, reference should be made to the narrative review by Ihle *et al.* and is not further discussed here (64).

Based on theories of muscle hypertrophy at the cellular level, Park *et al.* investigated the influence of blood flow restriction on increasing the effectiveness of postoperative training after HTO (65). They were able to show for a cohort of middle-aged women (45–65 years) that training under restriction of blood flow at 80% of arterial occlusive pressure (AOP) compared to training at 40% AOP and no restriction leads to superior results. Training at 80% AOP achieved significantly lower decrease in thigh muscles and higher strength values for knee extension after 12 weeks of training. The area of the thigh muscles was measured at two predefined points in axial MRI images, the maximum-arbitrary isokinetic contraction of the knee extensors was measured in an isokinetic dynamometer. The loading protocol was adapted to a 6-week phase of partial weight-bearing and a subsequent 6-week phase of full weight-bearing (65). These findings represent a possible treatment option in the long-term postoperative course. If the results are confirmed in further studies, this method can be integrated into the physiotherapeutic follow-up treatment.

#### Healthcare system characteristics

When evaluating postoperative treatment concepts hospitalization strategies should be considered with increasing interest. Up to now, most osteotomies seem to be performed in an inpatient setting that theoretically allow the application of the above presented procedures, but there are differences depending on the healthcare system, as well as an economically driven orientation towards more ambulatory surgeries in general. Doran *et al.* concluded in their retrospective work that knee osteotomies in terms of HTO, DFO and tibial tubercle osteotomy can be performed safely in an ambulatory setting (*n* = 222) without significant differences regarding readmission, reoperation or postoperative complications compared to hospitalized patients (*n* = 309) (66). Baseline characteristics showed no significant differences between the groups. Nevertheless, the study does not provide information on postoperative outcomes or rehabilitation protocols, as the follow-up only consisted of a data acquisition 90 days post operation. Therefore, no complications occurring after the 3 months period were recorded (66). Polacek *et al.* examined the outcome of 39 patients after a day-case HTO procedure using intraosseous PEEK (polyether ether ketone) implants with KOOS and found a steady improvement 6 and 12 months post operation (67). Complication rates were 18% in total and 10% revision rate. The study only has limited validity as there was no control group, but comparisons respectively to existing literature showed similar complication rates and results despite an average hospitalization time of 5.6 h (67). Except these studies with limited evidence levels, there are no larger studies discussing the feasibility or presenting results of knee osteotomies in an ambulatory setting. Given the mentioned trend towards ambulatory procedures, there is an urgent need for further evaluation in prospective, high evidence studies to maintain a high standard for patient safety and outcome. Consequently, postoperative rehabilitation protocols may differ or need adaption to an ambulatory setting. Also, different healthcare characteristics regarding supply and financing of above mentioned postoperative therapeutic options may influence rehabilitation protocols.

#### Current postoperative treatment protocol at the authors’ institution

At our clinic, patients are treated with MCS, IPC, CPM and anticoagulation after knee osteotomies. There is no standard use of tourniquets during osteotomy procedures. Depending on the type of osteotomy, an early functional weight-bearing protocol is pursued. After HTO with angular-stable plate osteosynthesis, 20 kg partial weight-bearing is prescribed for 2 weeks and then a transition to full weight-bearing under physiotherapeutic guidance is allowed. Distal femoral and double level osteotomies receive 20 kg partial weight-bearing without ROM limitation for 6 weeks. Furthermore, beginning on the first postoperative day, movement exercises and gait training are carried out on forearm crutches under physiotherapeutic guidance. After two to three days of hospitalization, physiotherapy is pursued by local therapists. According to the findings of this review, the use of MCS, CPM and IPC should be re-evaluated.

## Conclusion

This narrative review presents current scientific evidence regarding the postoperative treatment after knee osteotomies. This review underlines that there are currently no evidence-based postoperative treatment concepts. To optimize patient care, the postoperative period should be based on evidence-based, standardized treatment interventions. In order to develop an evidence-based postoperative treatment concept following knee osteotomies, well-designed high-quality studies must be conducted. As shown in this review, for some areas of postoperative treatment, such as postoperative weight-bearing protocols or pharmacological thromboembolism prophylaxis, studies with a high level of evidence or reviews are already available. For other sub-areas, such as methods for improved bone consolidation or mechanical thromboembolism prophylaxis, there are few or no high-evidence publications, systematic reviews are missing. In addition, the proportion of studies that examine lateral closed-wedge HTO and distal femoral osteotomies is significantly lower than studies that examine medial open-wedge HTO. The presented therapeutic options must be evaluated in the context of different healthcare system characteristics with varying supply and financing. The economic factor of postoperative treatment procedures has rarely been considered in publications. If there is evidence against improved treatment quality for a defined patient population for a procedure used, a cost–benefit evaluation must be carried out.

Overall, the postoperative treatment period following knee osteotomies is underrepresented in scientific discourse, especially with regard to the increasing popularity and implementation of the procedure in a young patient population.

## ICMJE Conflict of Interest Statement

The authors declare that there is no conflict of interest that could be perceived as prejudicing the impartiality of this review.

## Funding Statement

The authors acknowledge support from the Open Access Publishing Fund of the University of Tübingen.
